# Inequalities in healthy life expectancy by Federated States

**DOI:** 10.1590/S1518-8787.2017051000105

**Published:** 2017-06-01

**Authors:** Célia Landmann Szwarcwald, Dália Elena Romero Montilla, Aline Pinto Marques, Giseli Nogueira Damacena, Wanessa da Silva de Almeida, Deborah Carvalho Malta

**Affiliations:** I Instituto de Comunicação e Informação Científica e Tecnológica em Saúde. Fundação Oswaldo Cruz. Rio de Janeiro, RJ, Brasil; II Departamento de Enfermagem Materno Infantil e Saúde Pública. Escola de Enfermagem. Universidade Federal de Minas Gerais. Belo Horizonte, MG, Brasil

**Keywords:** Life Expectancy, Socioeconomic Factors, Health Inequalities, Health Surveys, Expectativa de Vida, Fatores Socioeconômicos, Desigualdades em Saúde, Inquéritos Epidemiológicos

## Abstract

**OBJECTIVES:**

To estimate the healthy life expectancy at 60 years by sex and Federated States and to investigate geographical inequalities by socioeconomic status.

**METHODS:**

Healthy life expectancy was estimated by the Sullivan method, based on the information of the National Survey on Health, 2013. Three criteria were adopted for the definition of “unhealthy state”: self-assessment of bad health, functionality for performing the activities of daily living, and the presence of noncommunicable disease with intense degree of limitation. The indicator of socioeconomic status was built based on the number of goods at household and educational level of the head of household. To analyze the geographical inequalities and socioeconomic level, inequality measures were calculated, such as the ratio, the difference, and the angular coefficient.

**RESULTS:**

Healthy life expectancy among men ranged from 13.8 (Alagoas) to 20.9 (Espírito Santo) for the self-assessment criterion of bad health. Among women, the corresponding estimates were always higher and ranged from 14.9 (Maranhão) to 22.2 (São Paulo). As to the ratio of inequality by Federated State, the medians were always higher for healthy life expectancy than for life expectancy, regardless of the definition adopted for healthy state. Regarding the differences per Federated State, the healthy life expectancy was seven years higher in one state than in another. By socioeconomic status, differences of three and four years were found, approximately, between the last and first fifth, for men and women, respectively.

**CONCLUSIONS:**

Despite the association of the mortality indicators with living conditions, the inequalities are even more pronounced when the welfare and the limitations in usual activities are considered, showing the necessity to promote actions and programs to reduce the socio-spatial gradient.

## RESUMO

**OBJETIVO:**

Estimar a esperança de vida saudável aos 60 anos por sexo e Unidade da Federação e investigar as desigualdades geográficas e por nível socioeconômico.

**MÉTODOS:**

A esperança de vida saudável foi estimada pelo método de Sullivan, com base nas informações da Pesquisa Nacional de Saúde, 2013. Foram adotados três critérios para definição de estado “não saudável”: autoavaliação de saúde ruim, funcionalidade para realização das atividades da vida diária, e presença de doença crônica não transmissível com grau intenso de limitação. O indicador de nível socioeconômico foi construído com base no número de bens no domicílio e grau de escolaridade do responsável. Para analisar as desigualdades geográficas e por nível socioeconômico, foram calculadas medidas de desigualdade, como a razão, a diferença e o coeficiente angular.

**RESULTADOS:**

A esperança de vida saudável entre os homens variou de 13,8 (Alagoas) a 20,9 (Espírito Santo) para o critério de autoavaliação de saúde ruim. Entre as mulheres, as estimativas correspondentes foram sempre mais altas e variaram de 14,9 (Maranhão) a 22,2 (São Paulo). Quanto à razão de desigualdades por Unidade da Federação, as medianas foram sempre maiores para a esperança de vida saudável do que para a esperança de vida, independentemente da definição adotada para estado saudável. Quanto às diferenças por Unidade da Federação, a esperança de vida saudável chegou a ser sete anos maior em um estado do que em outro. Por nível socioeconômico, foram encontradas diferenças de três e quatro anos, aproximadamente, entre os últimos e primeiro quintos, para homens e mulheres, respectivamente.

**CONCLUSÕES:**

Além de os indicadores de mortalidade estarem associados às condições de vida, as desigualdades são ainda mais pronunciadas quando o bem-estar e as limitações nas atividades habituais são levados em consideração, mostrando a necessidade de promover ações e programas para diminuir o gradiente socioespacial.

## INTRODUCTION

The portion of the world’s older population (aged 60 years or older) increased from 8% in 1950 to 13% in 2013^1^. Obeying to a faster pace of growth, the older population will represent 21% of the total in 2050^1^. With the aging of the population in both developing and developed countries, the noncommunicable diseases (NCD) became the most important health problem in most countries, generating disabilities and high degree of limitation of sick people in their work and leisure activities^2^.

The increased longevity in developed countries stressed the need for new health indicators that included quality measures^3^. Once a long life does not necessarily mean a healthy life, today, mortality indicators are considered insufficient to adequately characterize the state of health of a population^4^.

In Brazil, over the past 30 years, sharp demographic changes occurred. With the aging of the population, the Country experiences an epidemiological transition, with important changes in morbidity and mortality profile. The NCD have responded for more than 70% of deaths and loss of quality of life, with a substantial portion of the total burden of diseases attributed to the occurrence of the NCD among older people^5^. Recent information showed that one in two seniors have the diagnosis of at least one NCD^6^.

After the 2000s, different health indicators have been proposed to complement the studies on mortality, considering not only the profile of morbidity and mortality, but also the functional limitations^7^. In national surveys on health, the self-perception of health and the reported diagnosis of NCD have been widely used to establish differences in morbidity among population groups^8^. In addition, research has been focusing on the quality measurement of the last years of life and, aiming to assess the assistance given and prevention programs, it has used health indicators that combine mortality and morbidity data^9,10^.

Among the health indicators that consider information of morbidity and mortality in a single measure, the healthy life expectancy (HALE) obtained by the Sullivan method^11^ has been the most widely used indicator, by its simplicity and easy interpretation of results^12^. The HALE is a measure of the population health to estimate the expected number of “healthy years” for the individuals of a population in a given age. Definitions of “healthy” are generally based on self-assessment of health and presence of chronic disease or disability and functional or cognitive limitations^13^.

In Brazil, the HALE was estimated for the total adult population^9,12,14^, according to sex and age group, considering self-assessment of health, presence of chronic disease, or problems that limit their usual activities. However, the differences per Federated State (FS) in terms of healthy longevity have not been surveyed yet. Knowing that the different living conditions affect the regional pattern of morbidity and mortality in the Country^15^, this study aimed to investigate the socio-spatial inequalities of HALE by FS in the Brazilian older population.

## METHODS

The National Survey on Health (PNS) is a home-based and nationwide research held by the Brazilian Ministry of Health and by the Oswaldo Cruz Foundation, in partnership with the Brazilian Institute of Geography and Statistics (IBGE) in the years 2013 and 2014. The project was approved by the National Commission of Ethics in Research (Opinion 328,159, of 26 June 2013).

The sample of the PNS is a subsample of the *Amostra Mestra do Sistema Integrado de Pesquisas Domiciliares* (SIPD – Master Sample of Integrated Household Surveys) of IBGE. The subsample was selected by sampling by conglomerates in three stages, with stratification of the primary sampling units (census tracts). In the second stage, in each census tracts, a fixed number of households was randomly selected. In the third stage, in every household, a resident aged 18 years or older was selected with equiprobability^16^.

At the end of the fieldwork, 81,254 households were visited. Of these, 69,994 households were occupied. Exactly 64,348 household interviews and 60,202 individual interviews were conducted at the household with the selected resident.

The HALE is a measure of the population health that estimates the expected number of “healthy years” (years of life in good health) for the individuals of a population in a given age. Definitions of “healthy” are generally based on self-assessment of state of health, reported morbidity, and presence of functional or cognitive limitations^12^.

In this study, healthy life expectancy was estimated by Sullivan method^11^ in the older population (60 years or more) by sex and FS. The method consists of estimating the proportion of “years lived in good health” of the expected total number of years experienced by a cohort according to the mathematical equation presented by Romero et al.^12^. Life expectancy at 60 years by sex and FS was provided by IBGE^a^.

To establish the “unhealthy state,” three measures were used: self-assessment of health, functionality of older people to perform the activities of daily living, and the presence of at least one NCD with limitations resulting from the disease. The analysis of self-assessment of health was based on the following question of the individual questionnaire: “In general, how do you assess your health?” The answers ranged from 1 (very good) to 5 (very bad), which were aggregated into two categories: very good, good or regular; bad or very bad, corresponding, respectively, to the “healthy” and “unhealthy” states.

Regarding the functionality of the older adults, we used the indicator of functional limitation to perform activities of daily living (ADL): to eat alone, including holding a fork, cutting food and drinking from a cup; to shower alone, including getting in and out of the shower or bath; to go to the bathroom alone, including sitting and lifting the toilet; to get dressed alone, including putting on socks and shoes, closing the zipper, closing and opening buttons; to walk home alone from a room to another of the house; to lie down or get up of the bed without help. The variable corresponding to the case of the older adult having great difficulty (“cannot” or “has great difficulty”) to perform at least one of the ADL was used for the establishment of the “unhealthy state.”

As to the presence of NCD, the variable was composed by the answers to all questions regarding the diagnosis of chronic diseases, including hypertension, diabetes, heart disease, cerebrovascular accident (CVA), asthma, arthritis, chronic problem of column, work-related musculoskeletal disorder (WMSD), depression, other mental disease, lung disease, cancer, chronic kidney failure, or other chronic physical or mental illness not previously specified. In the case of affirmative response to any NCD, we asked questions about the degree of limitation to conduct the usual activities because of the disease. The indicator regarding the presence of at least one NCD with intense or very intense limitation degree to conduct usual activities was adopted to define the “unhealthy state.”

To analyze geographical inequalities of HALE at 60 years by FS, an indicator of socioeconomic status (SES) was considered, adapted from the Brazilian Association of Research Companies, using the number of goods at the household, educational level of the head of household, and presence of maid paid monthly. The SES indicator was used in the form of points obtained in each household, considering the mean by FS.

The outcomes considered in this study were life expectancy at 60 years, provided by IBGE; healthy life years at 60 years, calculated by the three definitions of healthy state; and total number of years lived with bad health perception. The following measures of geographical inequalities were used: ratio of inequalities, given by the ratio among the values found by FS and the minimum value; difference, given by the difference among the values found by FS and the minimum value.

As to inequality by SES, the following measures were used^17^: angular coefficient of inequalities, corresponding to the angular coefficient of the regression of each outcome with the SES indicator, ratio and difference among the estimates in the last and first fifth of the SES indicator. To estimate the angular coefficient of inequality, we used the statistical procedure of linear regression using the SES indicator as the independent variable. Angular coefficients and the values of the statistical significance level for each outcome were estimated.

Because it is a survey with stratification of primary sampling units and selection by conglomerates in three stages, the complex sampling design was considered in all statistical analysis of the data.

## RESULTS

Table 1 shows the variables used for the definition of the unhealthy state: proportion of older people with self-assessment of bad or very bad health, the proportion of seniors with some difficulty to carry out the activities of daily living, and proportion of seniors diagnosed with some NCD and intense or very intense degree of limitation and for carrying out usual activities because of the disease. The proportion of people with bad or very bad health self-assessment showed wide variation, from 2.5% to 24.0%, for males, and from 7.7% to 31.9%, for females, with maximum values found in Alagoas and Maranhão, respectively, and the minimum, in Espírito Santo and Rio de Janeiro, showing a clear North-South disparity.

**Table 1:** Proportion of older people in “unhealthy state” according to different definitions, per sex and State. National Survey on Health, 2013.

State	% bad SAH^a(1)^			% lim ADL^a(2)^			% NCD with lim^a(3)^			Mean of points^b^
		
	M	F	T	M	F	T	M	F	T	
Rondônia	14.1	12.5	13.3	5.7	5.8	5.7	12.3	12.5	12.4	17.4
Acre	12.5	14.1	13.3	6.4	5.1	5.7	10.5	15.8	13.2	15.2
Amazonas	12.2	13.3	12.8	4.9	6.5	5.8	9.4	10.6	10.0	16.9
Roraima	23.9	18.1	21.2	6.8	8.2	7.5	26.5	13.2	20.2	19.0
Pará	18.9	16.6	17.7	6.1	5.0	5.5	1.7	8.7	5.4	14.9
Amapá	16.2	11.5	13.6	5.6	5.6	5.6	14.5	8.1	10.9	17.4
Tocantins	17.5	19.5	18.4	6.0	6.8	6.4	13.7	32.9	22.9	16.8
Maranhão	18.9	31.9	25.7	8.5	9.0	8.8	9.6	16.3	13.1	13.5
Piauí	9.3	27.7	18.7	7.0	6.8	6.9	12.2	16.3	14.3	14.6
Ceará	8.9	15.3	12.5	5.9	7.0	6.5	10.5	17.0	14.1	16.7
Rio Grande do Norte	10.4	18.1	14.6	7.7	11.1	9.6	17.6	15.8	16.6	17.5
Paraíba	15.6	17.6	16.8	9.3	10.9	10.2	15.4	20.3	18.2	16.8
Pernambuco	20.0	19.1	19.5	8.0	10.9	9.7	10.0	14.3	12.4	17.9
Alagoas	24.0	23.7	23.8	8.1	12.0	10.3	13.6	9.8	11.5	16.1
Sergipe	12.0	12.3	12.2	5.8	9.2	7.7	11.8	12.4	12.1	16.9
Bahia	11.8	17.1	14.9	6.6	8.9	7.9	9.6	17.1	14.0	16.5
Minas Gerais	11.9	11.6	11.7	6.3	9.4	8.1	14.4	14.9	14.7	19.6
Espírito Santo	2.5	19.3	12.4	4.4	5.9	5.3	15.5	11.4	13.1	19.5
Rio de Janeiro	10.1	7.7	8.7	5.2	5.1	5.1	8.2	10.0	9.2	20.8
São Paulo	6.2	8.4	7.4	5.6	4.9	5.2	7.2	9.8	8.6	23.8
Paraná	19.6	13.4	16.2	8.1	8.3	8.2	14.1	18.0	16.2	22.2
Santa Catarina	7.8	17.6	13.3	4.2	5.0	4.7	10.6	19.6	15.6	23.1
Rio Grande do Sul	5.6	10.4	8.4	4.8	8.7	7.1	7.7	15.2	12.1	22.1
Mato Grosso do Sul	9.9	13.2	11.5	5.2	9.9	7.6	11.1	15.7	13.4	19.1
Mato Grosso	12.9	16.4	14.8	8.1	10.8	9.6	10.1	20.1	15.6	19.3
Goiás	15.3	13.6	14.4	5.8	11.2	8.8	14.6	25.7	20.7	20.1
Federal District	6.0	9.9	8.3	5.3	5.5	5.4	6.4	9.1	8.0	25.5
Brazil	10.9	13.0	12.1	6.1	7.3	6.8	10.0	13.8	12.2	19.9

M: male; F: female; T: total; SAH: self-assessment of health; Lim: limitation; ADL: activities of daily living; NCD: noncommunicable disease.

^a^ Unhealthy state definitions:

^(1)^ Self-assessment of bad or very bad health.

^(2)^ Has difficulty or is unable to perform the ADL.

^(3)^ Has at least one NCD with intense or very intense degree of limitation of usual activities because of the disease.

^b^ The mean was calculated by the number of points allocated to households of FS according to the number of goods of the household, educational level of the head of household, and the presence of monthly maid.

The proportion of seniors with some NCD and intense degree of limitation also varied substantially according to the FS. However, by relying on the diagnosis of chronic illness, the minimum and maximum values were found in the Northern States for both sexes. The indicator that showed lower values and the lowest amplitude of variation by FS was the proportion of people with some sort of functionality for ADL, with minimum values of 4.2% and 4.9%, and maximum of 9.3% and 12.0% between men and women, respectively (Table 1).

In Brazil, the lowest proportion of people living in an “unhealthy state” was obtained for the indicator of functionality issues, while the other two showed similar values. For any one of the three indicators considered, the proportion of women in “unhealthy state” is higher than among men (Table 1).

Table 1 also shows estimates of socioeconomic level indicator, calculated by the average number of points awarded to households of the FS. The minimum value was 13.5 in Maranhão and the maximum of 25.5 in the Federal District.

Table 2 shows the estimates of life expectancy at 60 years and HALE by sex according to the FS, considering the different definitions of healthy state. For males, life expectancy at age 60 ranged from 17.7 (Piauí) to 21.4 (Espírito Santo), and was 19.9 in Brazil. The HALE ranged from 13.8 (Alagoas) to 20.9 (Espírito Santo) for the self-assessment criterion of bad health, and from 13.5 (Roraima) to 19.2 (Federal District) for the presence of NCD with intense degree of limitation. For females, the corresponding estimates were always higher, and ranged from 20.0 (Roraima) to 25.4 (Espírito Santo), from 14.9 (Maranhão) to 22.2 (São Paulo), and from 14.8 (Tocantins) to 22.50 (Espírito Santo), respectively. Due to little variation in the proportion of older people with limitations of functionality by FS in both sexes, the HALE estimated with these criteria follows a sociogeographical pattern similar to that of life expectancy at 60 years.

**Table 2:** Estimates of life expectancy (LE) and healthy life expectancy (HALE) at age 60 according to the different definitions of “unhealthy state,” per sex and State. National Survey on Health, 2013.

State	LE^a^			bad HALE SAH^b(1)^			HALE lim ADL^b(2)^			HALE NCD with lim^b(3)^		
			
	M	F	T	M	F	T	M	F	T	M	F	T
Rondônia	17.9	20.6	19.1	15.4	18.0	16.6	16.9	19.4	18.0	15.7	18.0	16.8
Acre	19.4	22.6	20.9	17.0	19.5	18.2	18.1	21.5	19.7	17.3	19.1	18.2
Amazonas	18.5	21.6	20.0	16.2	18.7	17.4	17.6	20.2	18.8	16.8	19.3	18.0
Roraima	18.4	20.1	19.2	14.0	16.4	15.1	17.2	18.4	17.8	13.5	17.4	15.3
Pará	18.7	21.7	20.1	15.1	18.1	16.6	17.5	20.6	19.0	18.4	19.8	19.0
Amapá	19.9	22.6	21.3	16.7	20.0	18.4	18.8	21.4	20.1	17.0	20.8	18.9
Tocantins	19.7	22.1	20.8	16.2	17.8	17.0	18.5	20.6	19.5	17.0	14.8	16.1
Maranhão	18.0	21.8	19.9	14.6	14.9	14.8	16.4	19.9	18.2	16.2	18.3	17.3
Piauí	17.7	21.1	19.5	16.0	15.3	15.8	16.4	19.7	18.1	15.5	17.7	16.7
Ceará	19.5	22.5	21.1	17.8	19.0	18.5	18.4	20.9	19.7	17.5	18.7	18.1
Rio Grande do Norte	19.9	23.8	22.0	17.8	19.5	18.8	18.4	21.1	19.9	16.4	20.0	18.3
Paraíba	19.3	21.9	20.7	16.2	18.0	17.2	17.5	19.5	18.6	16.3	17.4	16.9
Pernambuco	18.5	21.8	20.3	14.8	17.7	16.4	17.0	19.4	18.4	16.6	18.7	17.8
Alagoas	18.1	21.7	20.0	13.8	16.5	15.2	16.6	19.1	17.9	15.7	19.5	17.7
Sergipe	18.3	21.8	20.2	16.1	19.1	17.7	17.2	19.8	18.6	16.1	19.1	17.7
Bahia	19.2	23.2	21.3	16.9	19.2	18.1	17.9	21.1	19.6	17.4	19.2	18.3
Minas Gerais	21.1	24.0	22.6	18.6	21.2	20.0	19.8	21.7	20.8	18.1	20.4	19.3
Espírito Santo	21.4	25.4	23.5	20.9	20.5	20.6	20.5	23.9	22.2	18.1	22.5	20.4
Rio de Janeiro	19.5	23.5	21.7	17.5	21.7	19.8	18.4	22.3	20.6	17.9	21.2	19.7
São Paulo	20.4	24.3	22.5	19.2	22.2	20.8	19.3	23.1	21.3	19.0	21.9	20.6
Paraná	20.4	23.5	22.0	16.4	20.3	18.4	18.7	21.5	20.2	17.5	19.2	18.4
Santa Catarina	20.9	25.1	23.1	19.2	20.7	20.0	20.0	23.9	22.0	18.6	20.2	19.5
Rio Grande do Sul	20.2	24.3	22.4	19.0	21.8	20.5	19.2	22.2	20.8	18.6	20.6	19.7
Mato Grosso do Sul	19.8	23.4	21.6	17.9	20.3	19.1	18.8	21.1	20.0	17.6	19.7	18.7
Mato Grosso	19.6	22.4	20.9	17.1	18.7	17.8	18.0	20.0	18.9	17.6	17.9	17.7
Goiás	19.7	22.2	20.9	16.7	19.1	17.9	18.6	19.7	19.1	16.8	16.5	16.6
Federal District	20.5	24.5	22.7	19.3	22.1	20.8	19.4	23.1	21.4	19.2	22.2	20.8
Brazil	19.9	23.4	21.8	17.7	20.4	19.1	18.6	21.7	20.3	17.9	20.2	19.1

M: male; F: female; T: total; SAH: self-assessment of health; Lim: limitation; ADL: activities of daily living; NCD: noncommunicable disease.

^a^ Brazilian Institute of Geography and Statistics (IBGE) – Full board of mortality in Brazil, 2013. Brief analysis of mortality in the periods 2012–2013 and 1980–2013. Research Directorate Coordination of Population and Social Indicators, 2014.

^b^ Estimates of the HALE using data of the National Survey on Health, 2013, by the Sullivan method according to the following definitions of unhealthy state:

^(1)^ Self-assessment of bad or very bad health.

^(2)^ Has difficulty or is unable to perform the ADL.

^(3)^ Has at least a grade or NCD with intense or very intense degree of limitation for usual activities because of the disease.

For the entire Country, the number of years lost by functional limitations in ADL was 1.2 among men, 1.7 among women, and 1.5 for the total. The highest number of lost years corresponded to the presence of NCD with intense degree of limitation because of the disease, 3.2 among women, and 2.0 among men. Similar results were found for the estimates based on self-perception of health.

Table 3 shows measures of geographical inequalities and by SES for the outcomes considered in the study. Regarding the ratio of inequality among the FS, the medians of ratio were always higher for the HALE than for life expectancy, regardless of the definition adopted for healthy state. The maximum ratio of inequality surpassed 1.5, both between male and female seniors, when the “unhealthy state” was defined by the bad self-assessment of health. As to the differences by FS, the HALE was seven years higher in one state than in another.

**Table 3:** Measures of geographical inequality and socioeconomic status (SES) in estimates of healthy life expectancy (HALE) at age 60 according to the different definitions of “unhealthy state” per sex. National Survey on Health, 2013.

Ratio of inequalities	Inequality measures by Federated State											

	LE^a^			HALE SAH^b(1)^			HALE lim ADL^b(2)^			HALE NCD with lim^b(3)^		
			
	M	F	T	M	F	T	M	F	T	M	F	T
Median	1.10	1.12	1.09	1.21	1.29	1.22	1.12	1.13	1.22	1.28	1.30	1.19
Maximum	1.21	1.26	1.23	1.51	1.49	1.41	1.25	1.30	1.25	1.42	1.52	1.36

**Differences**	**LE**			**HALE SAH**			**HALE lim ADL**			**HALE NCD with lim**		
			
	**M**	**F**	**T**	**M**	**F**	**T**	**M**	**F**	**T**	**M**	**F**	**T**

Median	1.85	2.37	1.81	2.91	4.27	3.31	1.93	2.46	1.82	3.81	4.41	2.84
Maximum	3.74	5.31	4.36	7.08	7.34	6.03	4.04	5.46	4.48	5.65	7.66	5.52

**Estimates**	**Estimates of LE and HALE by fifths of SES indicator^c^**											

	**LE**			**HALE SAH**			**HALE lim ADL**			**HALE NCD with lim**		
			
	**M**	**F**	**T**	**M**	**F**	**T**	**M**	**F**	**T**	**M**	**F**	**T**

1^st^ fifth	18.82	22.37	20.66	16.25	18.01	17.17	17.55	20.60	19.13	17.09	18.85	18.06
2^nd^ fifth	19.27	22.59	21.00	16.64	18.73	17.73	17.93	20.45	19.25	16.81	19.22	17.99
3^rd^ fifth	20.25	23.56	22.03	17.90	21.17	19.66	19.08	21.74	20.51	17.83	20.27	19.14
4^th^ fifth	20.39	24.19	22.39	18.11	21.09	19.69	19.19	22.29	20.83	18.22	20.08	19.21
5^th^ fifth	20.44	24.26	22.51	19.17	22.20	20.82	19.31	23.07	21.34	18.98	21.89	20.57

**Measures**	**Inequality measures by SES indicator**											

	**LE**			**HALE SAH**			**HALE lim ADL**			**HALE NCD with lim**		
			
	**M**	**F**	**T**	**M**	**F**	**T**	**M**	**F**	**T**	**M**	**F**	**T**

Ratio 1^st^/5^th^ fifths	1.09	1.08	1.09	1.18	1.23	1.21	1.10	1.12	1.12	1.11	1.16	1.14
Differences 1^st^/5^th^ fifths	1.62	1.89	1.84	2.92	4.20	3.65	1.75	2.47	2.21	1.89	3.04	2.51
Angular coefficient	0.24	0.30	0.28	0.37	0.52	0.45	0.26	0.31	0.30	0.24	0.30	0.27

M: male; F: female; T: total; SAH: self-assessment of health; Lim: limitation; ADL: activities of daily living; NCD: noncommunicable disease; LE: life expectancy.

^a^ Brazilian Institute of Geography and Statistics (IBGE) – Full board of mortality in Brazil, 2013. Brief analysis of mortality in the periods 2012–2013 and 1980–2013. Research Directorate Coordination of Population and Social Indicators, 2014.

^b^ Estimates of the HALE using data of the National Survey on Health, 2013, by the Sullivan method, according to the following definitions of unhealthy state:

^(1)^ Self-assessment of bad or very bad health.

^(2)^ Has difficulty or is unable to perform the ADL.

^(3)^ Has at least a grade or NCD with intense or very intense degree of limitation for usual activities because of the disease.

^c^ Number of points obtained in accordance with the goods at the household, educational level of the head of household, and monthly maid.

Considering the distribution of the SES indicator considering the older population of each FS, differences of three and four years were found, approximately, between the last and first fifth, for men and women, respectively, in the HALE estimated with the bad or very bad self-assessment criterion (Table 3).

Concerning the inequality coefficients by socioeconomic level, calculated by the angular coefficient of the regression models adjusted to four outcomes and having the SES indicator as independent variable, we observe positive and significant associations both in life expectancy and in HALE, for both sexes. In addition, when considering the self-assessment of bad health or functional limitations for the ADL for the definition of “unhealthy state,” the gradients by SES are more pronounced than those obtained for life expectancy at 60 years (Table 3 and Figure).

**Figure:**
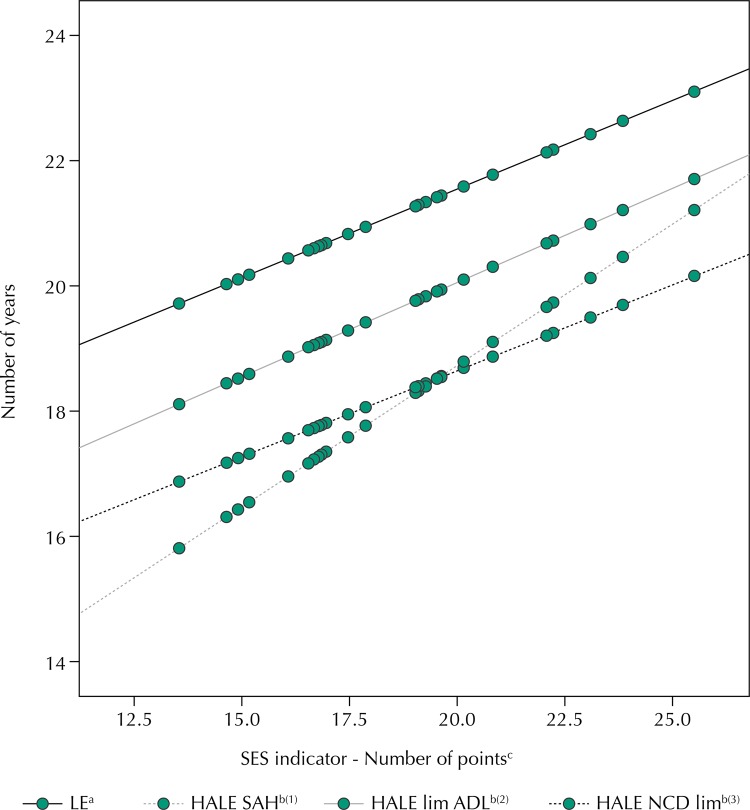
Predicted values of life expectancy (LE) and healthy life expectancy (HALE) according to the three definitions of “healthy state” and the indicator of socioeconomic status. National Survey on Health, 2013.

M: male; F: female; T: total; SAH: self-assessment of health; Lim: limitation; ADL: activities of daily living; NCD: noncommunicable disease.

^a^ Brazilian Institute of Geography and Statistics – Full board of mortality in Brazil, 2013. Brief analysis of mortality in the periods 2012–2013 and 1980–2013. Research Directorate Coordination of Population and Social Indicators, 2014.

^b^ Estimates of the HALE using data of the National Survey on Health, 2013, by the Sullivan method according to the following definitions of unhealthy state: ^(1)^ Self-assessment of bad or very bad health; ^(2)^ Has difficulty or is unable to perform the ADL; ^(3)^ Has at least one NCD with intense or very intense degree of limitation to conduct usual activities because of the disease.

^c^ Number of points obtained in accordance with the goods at the household, educational level of the head of household and monthly maid.

## DISCUSSION

This article is the first national study to show inequalities in healthy life expectancy by FS. Regarding the general aspects, the results were consistent with previous studies: although women live longer than men, they live relatively less years in good health^18^. In addition to the variation in life expectancy at birth, marked inequalities in healthy longevity were found among the states of more and less developed regions of Brazil.

To calculate the HALE, we used the Sullivan method, adopting three different criteria to define the unhealthy state. The first criterion was based on self-assessment of health, since the expanded concept of health transcends the absence of death, disease and disability, and incorporates concepts of welfare and quality of life. Unlike the medical assessment of the state of health, which identifies a disease by a set of signs, symptoms and laboratory data, the self-perception of health combines subjective, physical and emotional components of welfare^19^. The second criterion was based on the functionality of the older adults, being understood that the functional limitations to perform the daily activities constitute an important loss in quality of life. Finally, the third criterion was based on the occurrence of intense degree of limitation for carrying out the usual activities due to a NCD.

The results showed high ratio of inequality of HALE at 60 years by FS, always higher than those obtained for the life expectancy, regardless of the indicator used for the definition of a healthy state. For the HALE estimated based on bad perception of their own health, the ratio of inequalities reached values above 1.50, meaning that the expected number of years lived in good health by the older population of a given state can be 50% higher than that of other state in Brazil.

Regarding inequality coefficients of HALE according to the variable representative of SES, the gradients were all significant, both for men and women, and more pronounced than those obtained for life expectancy, with the exception of estimates calculated by the presence of intense limitation because of some NCD. In the context of the comparison of estimates of HALE by SES, unhealthy state definitions based on morbidity do not work well, because they depend on the access to diagnosis, admittedly uneven by region and area of residence (urban/rural)^20^.

With the current growth of longevity experienced by populations around the world, the proportion of unhealthy years also tends to increase, and the measures of healthy longevity becomes even more important. Different techniques have been proposed to refine the simple binary measures of state of health, such as the welfare composite index from Canada based on eight areas of health^21^. In the case of the HALE calculated with the self-assessment of bad health, for example, the differences by SES reflect not only the influence of the sociostructural determinants, but also the consequences of having a health problem in areas socially disadvantage^18^. Despite this limitation, the recurring use of self-perception of health comes from its validity, established by its association with objective measures of health problems^6^. In this study, the HALE calculated based on bad self-assessment was the most sensitive to show both geographical inequalities, such as socioeconomic status.

Effects of socioeconomic inequalities on healthy longevity have been highlighted in national and international studies, with results invariably worse in socially disadvantaged groups. In Rio de Janeiro, among people aged 65 years or older, the HALE was two times lower in the sector with the highest concentration of slum population than in the richest area of the city^22^. In England, in a study that combined health-related quality of life indicators with mortality data, a difference of 11 years on HALE was found between the worst and the best fifth socioeconomic status^23^. Comparative analysis of HALE at 50 years in European countries from 2005 to 2010 showed growing inequality, explained, possibly by the worsening in living conditions and increased unemployed^24^.

One of the limitations of this study concerns the sample size of the PNS among individuals aged 60 years or older, insufficient for the estimation of HALE by age group, for example, at the age of 70 years. In addition, data on income of the PNS have not yet been disclosed, restricting the studies on inequality by socioeconomic level to the indicator built from the goods at household and the level of education of the head of household.

With the results of this study, we concluded that not only the mortality indicators are associated with living conditions, but that inequities are even more pronounced when the welfare and the limitations in usual activities are considered, pointing to the necessity to promote actions and programs to decrease the socio-spatial gradient. In the current epidemiological context, the development of local strategies is essential not only to provide assistance to all people in need of care, but also to support the policies of prevention and the adoption of healthy behaviors, essential to achieve longevity with quality.

## Footnotes

a Instituto Brasileiro de Geografia e Estatística. Tábua completa de mortalidade para o Brasil – 2013. Breve análise da mortalidade nos períodos 2012-2013 e 1980-2013. Diretoria de Pesquisas. Coordenação de População e Indicadores Sociais, 2014.
